# Neural coding tools, based on Information Theory, applied to discrete time series: from electrophysiology to neuroethology

**DOI:** 10.1186/1471-2202-12-S1-P253

**Published:** 2011-07-18

**Authors:** Caroline Garcia Forlim, Ludmila Brochini Rodrigues, Reynaldo Daniel Pinto

**Affiliations:** 1 Instituto de Física, Universidade de São Paulo,São Paulo 05508-090 SP, Brazil; 2 Instituto de Física de São Carlos, Universidade de São Paulo, São Carlos 13560-970 SP, Brazil

## 

We developed a method based on Information Theory [1] to the analysis of discrete time series applied to spike and pulse sequences in two different systems: Central Pattern Generator (CPG) motor neurons in the crustacean stomatogastric ganglion (biological and model neurons) and electrocommunication in weakly electric fish interacting with an artificial fish.

CPG motor neurons [4][5] in the nervous systems are responsible for the mechanical digestion flow of fluids from the stomach to the intestines. They present an extensive dynamic repertoire due to a large variety of ion channels and modulation mechanisms. The rhythm and the average discharge frequency of these neurons must be constant to ensure a proper muscular contraction, nevertheless there are patterns associated to a very detailed inter spike distributions in which muscular contraction do not take part [3]. What are these patterns for? Experiments were performed in a pair of pyloric CPG neurons with simultaneously intracellular recordings, both in control condition (intact CPG) and with a hybrid network where one neuron was replaced by a computational model (real time interface). Information theoretical analysis showed that a motor neuron is able to express information encoded in spike intervals (ISIs) from another one (both biological and artificial neuron) in a very fine time scale.

Weakly electric fish provide a wonderful opportunity to study and interact with a living intact nervous systems. Their electrical organ discharges (EODs) are not only complex signals that belong to the processing of environmental sensory information by the nervous system but they are also easy-to-detect. Each EOD generates an electric field that reaches a myriad of electroreceptors disposed to detect small changes in the cutaneous electric field. With this apparatus the weakly electric fishes are able to detect objects as well as social electrocommunication[2]. Using non invasive techniques in a freely moving fish we were able to record its organ electric discharges (EODs) for long periods (~ hours). Electrocommunication experiments were performed, in which electric stimuli sequences, mimicking the fish EODs, were sent to a measurement tank *via* artificial fish, both in real time and previously recorded sequences. Analysis using Information Theory tools revealed that the stimuli sequences play a significant role in the fish response.

## References

[B1] Borst A Theunissen FE Information theory and neural coding Nat Neurosci 1999 2 947 957 10.1038/14731 10526332

[B2] Bullock TH Hopkins CD Popper AN Fay RR Eletroreception 2005 1 Springer

[B3] Hooper SL Gucshlbauer V von Uckermann G Büschges A Different motor neuron spike patterns produce contractions with very similar rises in graded slow muscles J Neurophysiol 2007 97 1428 1444 10.1152/jn.01014.2006 17167058

[B4] Selverston AI Invertebrate central pattern generator circuits Philos Trans R Soc Lond B Biol Sci 2010 365 2329 45 10.1098/rstb.2009.0270 20603355 PMC2894947

[B5] Selverston AI Russell DF Miller JP The stomatogatric nervous systems: structure and function of a small neural network Prog Neurobiol 1976 7 215 290 10.1016/0301-0082(76)90008-3 11525

